# Prosodic Focus Marking in Silent Reading: Effects of Discourse Context and Rhythm

**DOI:** 10.3389/fpsyg.2016.00319

**Published:** 2016-03-08

**Authors:** Gerrit Kentner, Shravan Vasishth

**Affiliations:** ^1^Department of Linguistics, Goethe UniversityFrankfurt, Germany; ^2^Department of Linguistics, University of PotsdamPotsdam, Germany

**Keywords:** linguistic rhythm, focus, accent, reading, implicit prosody, syntactic parsing, sentence comprehension, eye tracking

## Abstract

Understanding a sentence and integrating it into the discourse depends upon the identification of its focus, which, in spoken German, is marked by accentuation. In the case of written language, which lacks explicit cues to accent, readers have to draw on other kinds of information to determine the focus. We study the joint or interactive effects of two kinds of information that have no direct representation in print but have each been shown to be influential in the reader's text comprehension: (i) the (low-level) rhythmic-prosodic structure that is based on the distribution of lexically stressed syllables, and (ii) the (high-level) discourse context that is grounded in the memory of previous linguistic content. Systematically manipulating these factors, we examine the way readers resolve a syntactic ambiguity involving the scopally ambiguous focus operator *auch* (engl. “too”) in both oral (Experiment 1) and silent reading (Experiment 2). The results of both experiments attest that discourse context and local linguistic rhythm conspire to guide the syntactic and, concomitantly, the focus-structural analysis of ambiguous sentences. We argue that reading comprehension requires the (implicit) assignment of accents according to the focus structure and that, by establishing a prominence profile, the implicit prosodic rhythm directly affects accent assignment.

## 1. Introduction

What are the factors determining the syntactic analysis of written text and how do they interact? The vast literature on written sentence comprehension suggests that readers make use of a multitude of information sources in order to extract structure from the printed letter string and compute its meaning. Some of these sources are represented directly in print, e.g., the words that contribute their meanings, or the punctuation that marks the partitioning of phrasal chunks. Other kinds of information have to be derived or inferred from the reader's linguistic and world knowledge. In making such inferences, the reader forms interpretations that constitute predictions about the upcoming text. These predictions may or may not turn out to be compatible with the actual structure of the sentence. The ease with which a reader traverses a text is based to a great extend on how accurate his predictions are.

In this study, we will be concerned with the interaction of two information sources that (i) tap the reader's linguistic knowledge, (ii) have no direct representation in print, and (iii) have each been shown to be influential in the reader's text comprehension process. One of these is the discourse representation (hereafter, context) which is based on the memory of previous linguistic content. The other more local type of information concerns the prosodic structure, specifically the linguistic rhythm that emerges from the succession of lexically strong and weak syllables. The results of two reading experiments presented here attest that discourse context and local linguistic rhythm, two otherwise independent phenomena, conspire to guide the syntactic analysis of structurally ambiguous sentences.

### 1.1. Implicit prosody and discourse context in reading

There is hardly any doubt that readers generate a mental prosodic-phonological representation of written texts even in silent reading (Chafe, 1988; Frost, 1998; Ashby and Clifton, 2005; Ashby and Martin, 2008; Savill et al., 2011) and a growing body of evidence supports the idea that these representations, conventionally called *implicit prosody* (Fodor, 2002), co-determine the way in which syntactic ambiguities are resolved (e.g., Bader, 1998; Hirose, 2003; Jun, 2003; Hwang and Steinhauer, 2011), see Breen (2014) for a review. In our own work, we found that readers, when faced with syntactically ambiguous structures, avoid interpretations the phonological representation of which involves a stress clash (i.e., a sequence of two adjacent syllables bearing lexical or post-lexical stress), and instead favor syntactic alternatives that allow for more felicitous, alternating rhythm of strong and weak syllables (Kentner, 2012, 2015; McCurdy et al., 2013). Similarly, Breen and Clifton (2011, 2013) provide evidence for very early prosodic effects contributing significantly to reading effort in ambiguous sentences when the reanalysis of the part-of-speech in noun-verb homographs involves a change in lexical stress [e.g., abstract (noun) vs. abstract (verb)]. These studies suggest that representations of lexical stress and the expectation of rhythmically alternating syllabic structure not only reflect but potentially direct readers' syntactic parsing decisions.

As for the role of contextual information in sentence comprehension, it has been shown extensively that the previous discourse may bring about strong expectations that guide the syntactic analysis: relevant information in the context may lead to the cancellation of otherwise strong garden path effects (e.g., Altmann and Steedman, 1988; Spivey and Tanenhaus, 1998; Binder et al., 2001; Snedeker and Trueswell, 2004). This has been taken as key evidence in favor of models embodying a multitude of potentially competing information sources simultaneously constraining the way in which the sentence is analyzed (cf. MacDonald et al., 1994; McRae et al., 1998; van Gompel et al., 2001).

Yet, while the influence of both implicit prosody and context on syntactic parsing are each attested, it remains largely unclear whether and how exactly these two kinds of constraint interact in guiding the parsing process. We are aware of two studies that explore the effects of both implicit prosody and discourse context in reading. The first one by Stolterfoht et al. (2007) uses ERP to study the processing of a certain type of ellipsis, so-called replacives (Drubig, 1994), in which a stranded argument is contrastively related to an argument in the preceding main clause (the correlate) (1).

(1) Am Dienstag hat …
     On  Tuesday  has  …
  a. der      Rektor   (nur)   [den   Schüler]*_F_* getadelt,
       the_*nom*_ principal (only) the_*acc*_ pupil          critizised,
       nicht [den Lehrer]_*F*_.
       not   the_*acc*_ teacher.
       *On Tuesday, the principal criticized the pupil, not the teacher.*
  b. (nur)  [der      Rektor]*_F_*  den     Schüler getadelt,
       (only) the_*nom*_ principal    the_*acc*_ pupil     critizised,
       nicht [der      Lehrer]_*F*_.
       not   the_*nom*_   teacher.
       *On Tuesday, the principal, not the teacher, criticized the pupil.*

In the study by Stolterfoht et al. (2007), the morphological case of this stranded, sentence-final argument determines which argument in the main clause is contrastively focussed and, correspondingly, accented—viz. the one bearing the same morphological case. With the presence or absence of the focus particle *nur* (“only”), Stolterfoht et al. (2007) varied the need for the reader to revise the default reading with wide focus to a narrow focus reading, and—depending on the association of *nur* with the subject or the object, they manipulated the need for revising the implicit accent placement when encountering the replacive argument. The ERP results suggest that these two processes—restructuring of focus domains and reassignment of (implicit) accentuation—are independent as they engender different ERP signatures. However, the study remains inconclusive as to whether and how focus structure and implicit accent placement interact in determining a syntactic analysis of the written sentence.

McCurdy et al. (2013) studied the effects of implicit prosody and contextual bias on syntactic parsing using eye-tracking methodology. Building on Bader (1996) and Kentner (2012), the target sentence involves an ambiguity concerning the word sequence *nicht mehr* which could either be resolved as a temporal adverb or as a negated comparative quantifier. In the latter case, *mehr* is accented in a spoken rendition of the sentence, while the temporal reading engenders main phrasal accent on the following verb. In their study, McCurdy et al. (2013) presented readers with a context sentence that was devised so as to bias for one specific reading of the subsequently presented ambiguous target sentence—a manipulation akin to syntactic priming (cf. the boxed portions in the context in (2), corresponding to comparative or temporal adverbs, respectively). As in Kentner (2012), the target sentence was subject to prosodic manipulations concerning the distribution of lexically stressed and unstressed syllables in the ambiguous region; this manipulation lead to either a rhythmically alternating sequences of lexical stresses or to stress clash, depending on the syntactic analysis, which in turn determined the (implicit) accentuation of the ambiguous word *mehr*.

(2) *Context*
         Comparative:
             Der Manager verlangt von Peer, 
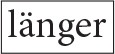
 zu trainieren, 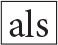 alle anderen.
             *The manager expects Peer to train longer than all the others.*
         Temporal:
             Peers Manager hat leider 
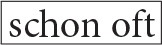
 zuviel von Peer gefordert.
             *Peer's manager has often asked too much of Peer*.
         *Target*
             Peer denkt, dass der Trainer…
             *Peer thinks that the trainer…*
         Temp… nicht mehr {zulassen/erlauben} sollte, dass er so viel trainiert.
                   *…should not {permit/allow} anymore that he trains so much.*
         Comp…  nicht mehr {zulassen/erlauben}  sollte, als tägliches Training.
                   *…should not {permit/allow} more than daily training.*

Replicating findings by Kentner (2012), the results reveal that the readers' avoidance of stress clash configurations significantly contributed to their parsing decisions. This effect was detectable already before the readers' eyes made contact with the disambiguating region. Effects of context on syntactic ambiguity resolution affected later parsing stages only and there was hardly any interaction between these information sources in the eye-movement record.

To summarize, the current state of affairs suggests that, if at all, local linguistic rhythm and more global discourse context interact only weakly, with local prosodic effects preceding any effects of the contextual manipulation. Although contextual information has been reported to affect the earliest stages of sentence comprehension, preferences from more local information sources have been claimed to potentially override contextual biases (Pickering and van Gompel, 2006). This may in fact explain the relatively late influence of context reported in McCurdy et al. (2013). An alternative explanation for the late effect of the contextual manipulation is the non-compelling nature of the bias that was introduced in McCurdy et al. (2013). In contrast to other studies probing contextual influences on syntactic parsing (e.g., Altmann and Steedman, 1988), McCurdy et al. (2013) did not aim at directly manipulating discourse representations. Rather, the context merely anticipated one of the morpho-syntactic structures of the ambiguous target sentence to create a bias for the corresponding interpretation.

### 1.2. The prosody and syntax of the focus particle *auch*

To specifically address the interplay of discourse representations and implicit prosody in sentence comprehension, we set out to study a different kind of syntactic ambiguity, the proper resolution of which hinges on contextual information. The ambiguity concerns the interpretation of the focus particle *auch* (engl.: “also”) in German (cf. Altmann, 1976; Jacobs, 1983; Sudhoff, 2008; Féry, 2009). Consider the ambiguous example in (3) with the three presuppositional interpretations in (3-a), (3-b), and (3-c). In writing, (3) is ambiguous with respect to the scope of *auch*, which may associate with either subject focus or object or VP-focus^1^. In the oral rendition, the ambiguity is (partly) resolved by prosody: unaccented *auch* and nuclear accent (the most prominent pitch accent in a sentence) on the object *Keller* presupposes that other objects beside the one stated are being rummaged through—hence, *auch* associates with focus on the object *Keller* or on the whole VP *Keller durchstöbert*. The interpretations with object focus and VP-focus have comparable prosodic renderings. Conversely, a rendition with accent on *auch* and a deaccented VP (*Keller durchstöbert*) presupposes that the object, in fact the whole VP, is outside the focus induced by *auch*; this accented rendition of *auch* leads to the presupposition that another person in addition to *Herbert* is the agent of the event expressed in the predicate (*auch* associates with the subject and, consequently, the subject is focussed).

(3)      Sonja meint, dass Herbert auch Keller durchstöbert.
        lit.: Sonja thinks that Herbert also cellars rummages.
        *Sonja thinks that Herbert(, too,) is rummaging through cellars(, too).*
  a. *H. is rummaging through something in addition to cellars*.
       (object focus)
  b. *H. is doing something in addition to rummaging through cellars.*
        (VP focus)
  c. *Somebody apart from H. is rummaging through cellars.*
        (subject focus)

A preceding context that renders the object in the target sentence either discourse-new or given restricts the room for interpretation to one of the possible interpretations. That is, a context like (4-a) makes the object *Keller* in (3) a new discourse entity. Hence, it is only compatible with *auch* associating with object focus. In that case, nuclear accent is required on the focused object, leaving *auch* prosodically unaccented. In contrast, in (4-b), the whole VP including the object *Keller* is explicitly mentioned. According to the mapping rules concerning information structure and prosody (e.g., Gussenhoven, 1983; Ladd, 1996; Vallduví and Engdahl, 1996; Schwarzschild, 1999; Féry and Samek-Lodovici, 2006; Krifka, 2006; Truckenbrodt, 2006), the givenness of the VP induces its deaccentuation and *auch* becomes the locus of nuclear accent (Féry, 2009), thus signaling association with focus on the subject. That is, when preceded by a relevant context, the ambiguity in (3) is properly resolved on the object. Its information status (new or given) unequivocally determines the syntactic association of the focus particle, and, correspondingly, the position of the accent.

(4)     Herbert und Karlo sammeln alte Möbel    für den
            Herbert and Karlo collect    old furniture for the
            Flohmarkt.
            fleamarket.
  a.    Karlo durchstöbert        Garagen.
           Karlo rummages through garages.
           *Karlo is rummaging through garages.*
  b.    Karlo durchstöbert        Keller.
           Karlo rummages through cellars.
           *Karlo is rummaging through cellars.*

## 2. Experiments

### 2.1. General design

To probe the interaction of implicit prosodic rhythm and contextual information, we applied a a 2 × 2 × 2 factorial design with two rhythmic factors crossed with the above contextual variation, which induces either subject or object focus in the target sentence. First, for the rhythmic context to the left of the ambiguous *auch* (RhythmLeft), the lexical material of the target sentences was constructed to yield a trochaic beat with every other syllable bearing lexical stress. The syllabic structure of the proper name directly preceding *auch* was systematically varied, with either a monosyllable or a disyllabic trochee [contrast between conditions a,b,c,d (trochaic name) vs. e,f,g,h (monosyllabic name) in (5)]. The logic of RhythmLeft is based on evidence for rhythmic entrainment (e.g., Dilley and McAuley, 2008; Niebuhr, 2009; Schmidt-Kassow and Kotz, 2009, w.r.t. auditory linguistic rhythm). If the proper name preceding ambiguous *auch* is trochaic, i.e., ends in an unstressed syllable (conditions a,b,c,d), *auch* falls onto a strong position of the beat established by the preceding word string and is thus more likely to receive prosodic prominence in the form of a (nuclear) accent. Conversely, if preceded by a monosyllabic word, *auch* would be in off-beat position which is predicted to hamper assignment of prosodic prominence.

The rhythmic environment to the right (RhythmRight) is manipulated on the object noun with lexical stress falling either on the initial or onto the second syllable (contrast between conditions a,c,e,g vs. b,d,f,h). An object bearing initial stress leads to a stress clash when the preceding *auch* is prosodically prominent. An iambic object, featuring an unstressed initial syllable, leads to a stress lapse when *auch* remains unaccented.

That is, depending on the accentuation of *auch* as determined by the discourse context, the rhythmic manipulations lead to alternating sequences of stressed and unstressed syllables or to phonologically unsatisfactory clashes or lapses in the context of *auch*. On the basis of our previous studies (Kentner, 2012; McCurdy et al., 2013), we assume that readers favor syntactic parses whose phonological representation has a favorable rhythm. Reading difficulties are predicted to emerge when the contextual manipulation forces a syntactic parse with rhythmically deviant prosodic structure.

(5)    I     Sonja Kohn und Herbert Otten sind bei einer Sicherheitsfirma angestellt.
                 Klaus hat erfahren, dass Sonja Kohn Kollegen überwacht.
                 *Sonja Kohn and Herbert Otten work for a security company*.
                 *Klaus has learned that Sonja Kohn supervises colleagues.*
                 a      Carla  glaubt,   dass   Herbert   Otten   auch Kollegen überwacht.
                         *Carla thinks that Herbert Otten supervises colleagues, too.*
                         (SubjFoc;  RhythmL=on beat;  RhythmR=no  Clash)
                 b      Carla  glaubt,   dass   Herbert   Otten   auch Lehrlinge überwacht.
                         *Carla thinks that Herbert Otten supervises apprentices, too.*
                         (ObjFoc;      RhythmL=on      beat; RhythmR=Clash)
         II     Sonja Kohn und Herbert Otten sind bei einer Sicherheitsfirma angestellt.
                Klaus hat erfahren, dass Sonja Kohn Lehrlinge überwacht.
                 c      Carla  glaubt,   dass   Herbert   Otten   auch Kollegen überwacht.
                          (ObjFoc; RhythmL=on beat; RhythmR=no Clash)
                 d      Carla  glaubt,   dass   Herbert   Otten   auch Lehrlinge überwacht.
                          (SubjFoc;    RhythmL=on    beat;    RhythmR=Clash)
         III    Sonja Kohn und Herbert Ott sind bei einer Sicherheitsfirma angestellt.
                 Klaus hat erfahren, dass Sonja Kohn Kollegen überwacht.
                 e      Carla glaubt, dass Herbert Ott auch Kollegen überwacht.
                          (SubjFoc; RhythmL=off beat; RhythmR=no Clash)
                 f       Carla glaubt, dass Herbert Ott auch Lehrlinge überwacht.
                          (ObjFoc;    RhythmL=off    beat;    RhythmR=Clash)
         IV    Sonja Kohn und Herbert Ott sind bei einer Sicherheitsfirma angestellt.
                 Klaus hat erfahren, dass Sonja Kohn Lehrlinge überwacht.
                 g      Carla glaubt, dass Herbert Ott auch Kollegen überwacht.
                         (ObjFoc; RhythmL=off beat; RhythmR=no Clash)
                 h      Carla glaubt, dass Herbert Ott auch Lehrlinge iiberwacht.
                         (SubjFoc;    RhythmL=off    beat;    RhythmR=Clash)

### 2.2. Experiment I: unprepared oral reading

The first experiment concerns the effects of linguistic rhythm and discourse context on the prosodic realization of the eight conditions (5) in spontaneous (unprepared) oral reading. Based on previous experience with this design (Kentner, 2012, 2015), we make the following assumptions: in (unprepared) oral reading, the prosodic realization reflects the interpretation assigned to the ambigous *auch*, i.e., when speakers accent *auch* (i.e., deaccent the object), they take it to associate with subject focus, otherwise with object or VP focus^2^.

#### 2.2.1. Materials, participants, procedure

Twenty-four item sets like (5) were developed. The items were distributed over eight lists such that items and conditions were counterbalanced across the lists with each list containing exactly one condition from each item set. Additionally, each list contained 64 filler items from four unrelated experiments and three practice items not connected to any of the experimental items, yielding a total of 91 items. With the exception of the three initial practice items, the item order was determined by pseudo-randomization (van Casteren and Davis, 2006) (for each participant individually) such that items from the same experiment had a minimal distance of two intervening items from other experiments and items from the same experimental condition were separated by at least three fillers.

Twenty-four members (19 female) of the Goethe-University community (Frankfurt, Germany) took part in the experiment. All participants were native speakers of German with normal or corrected-to-normal vision per self report. Participants were not informed about the purpose of the experiment before the experiment began; they were debriefed after the experiment ended. The age range was between 19 and 50 years old.

The experiment took place in a silent office at Goethe University in single sessions for each participant. Participants were seated in front of a 21.5-inch computer screen and equipped with a microphone head set (Shure) attached to an R-44 digital recorder.

All 91 items of each list were presented in a coherent slide show created with the standard settings of the Latex beamer package (Tantau et al., 2015). Each item was presented on two consecutive screen displays. The first display presented the two context sentences in the upper half and the first two words of the target sentence (in the case of this experiment: subject and verb of the matrix clause) in the middle of the screen (all text left-aligned). Upon pressing the enter button on the keyboard, the target sentence appeared in full (leaving the rest of the first display intact). Participants were asked to read the first display (i.e., the context) silently before moving on to the second display screen. To ensure spontaneous, unprepared oral reading and minimal look-ahead, participants were instructed to read out the target sentence immediately as it appeared on screen and to do so as fluently as possible. The participants were discouraged from making corrections during or after reading and to move on to the next item after reading by another button press. The productions of the participants were recorded on a digital memory card.

#### 2.2.2. Results

All in all, (24 × 24=) 576 experimental sentences were recorded.

Two judges independently evaluated each target sentence. Their task was to determine by ear (i) whether the production was a fluent and flawless response to the target sentence, and (ii) where the nuclear accent was realized (on *auch* or on the object). In order to avoid influencing their judgment, the judges were not informed about the context that preceded the target sentence. Correspondingly, context-target inconsistencies with respect to the accentuation of *auch* were not coded as flaws.

Twenty-six sentences were scored as non-fluent or flawed by at least one judge. For 12 additional sentences, judges were unsure or did not agree as to where the nuclear accent was realized. These 38 sentences (6.6%) were discarded from further analysis.

**Figure 1:**
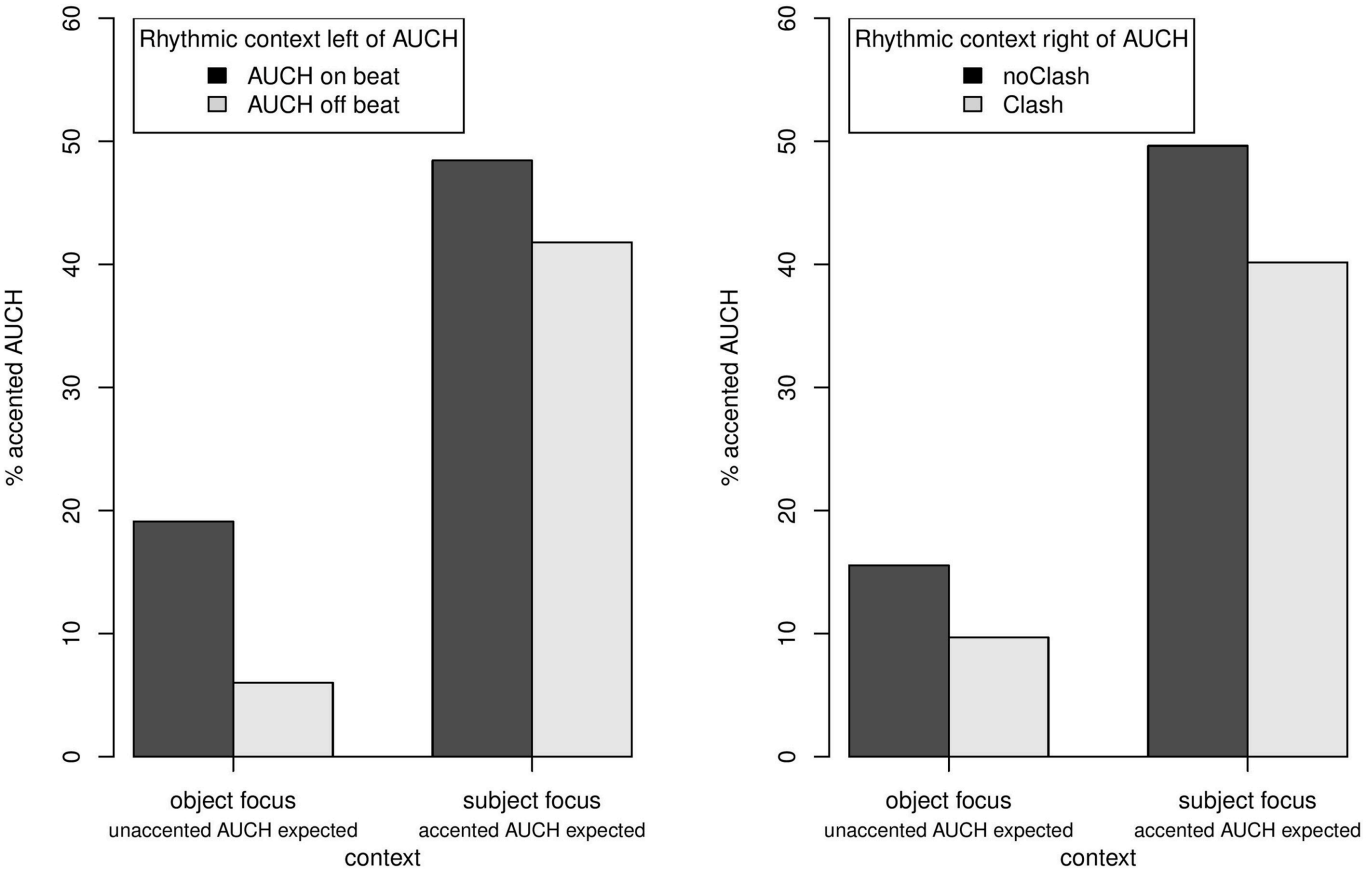
**Percentages of accented ***auch*** broken down by context (in both panels, the left pair of bars represent object focus, the right pair of bars represent subject focus) and rhythmic environment to the left (left panel: ***auch*** on beat vs. off beat) and rhythmic environment to the right (right panel: initial vs. non-initial stress on object)**.

Aggregating the 538 valid responses, *auch* was perceived as accented in about 24% of the cases (Figure 1). Note, that the full consideration of the context by the participants would imply 50% accented *auch* (all contexts inducing subject focus); correspondingly, only 67% of all trials were realized in accordance with the contextual conditions. We applied logistic mixed models (Bates et al., 2014) in the statistical computing environment R (R Core Team, 2014) to assess the effects of Context, RhythmLeft and RhythmRight as well as their interactions on the realization of accent. Accent was treated as a categorial variable (accent on *auch*: 1, accent on the object: 0). The fixed effects were coded as orthogonal sum contrasts to ensure minimal correlation; the contrast coding is shown in Table 1.

**Table 1:** **The contrast coding used for the statistical analyses**.

**Context**	
−1: accent unexpected	1: accent expected
(object focus)	(subject focus)
**RhythmLeft**	
−1: “auch” on beat	1: “auch” off beat
**RhythmRight**	
−1: non-initial stress on object	1: initial stress on object
(no clash)	(clash)

Random intercepts were included for participants and items. The results of the logistic mixed model are tabulated in Table 2. Over and above the preference for unaccented *auch*, the preceding Context significantly affected the realization of accent on *auch*. As expected, *auch* is more often accented when the preceding context renders the object given (accentuation of *auch* in 41% of cases), than when the object is new (accentuation in 8% of the cases). RhythmLeft has a weaker but still significant effect: *auch* is more likely to be accented when it falls onto the beat that is established by the rhythmic context to the left (*auch* accented in 28% of the cases) than when it is in off-beat position (20% accented).

**Table 2:** **Results of logistic mixed model on perceived accentuation of ***auch*** in experiment I (unprepared reading)**.

**Fixed effects:**	**Estimate**	**Std. Error**	**z value**	**Pr(>|z|)**
Context	1.27759	0.15913	8.029	<0.001
RhythmL	−0.30785	0.15236	−2.021	0.0433
RhythmR	−0.08269	0.15223	−0.543	0.5870
Context:RhythmL	0.15835	0.15199	1.042	0.2975
Context:RhythmR	−0.01391	0.15203	−0.092	0.9271
RhythmL:RhythmR	0.22844	0.15232	1.500	0.1337
Context:RhythmL:RhythmR	−0.35593	0.15263	−2.332	0.0197

The effect of RhythmRight on accentuation is not significant by itself but a significant three-way interaction Context:RhythmL:RhythmR attests the expected avoidance of stress clash (preference for leaving *auch* unaccented when the following syllable is stressed) in subject focus contexts when *auch* is in off-beat position, and in object focus contexts when *auch* is on-beat.

#### 2.2.3. Phonetic realization of accented vs. unaccented *auch*

As mentioned above, perceived accentuations of the target word *auch* are comparatively rare, i.e., in only about 24% of the cases. However, accentuation would be required in all subject focus contexts, i.e., 50% of the cases. The reason for this discrepancy is most likely due to the task (unprepared oral reading) and the general preference for function words to remain unaccented (Bader, 1996).

In order to exclude misperception by the judges as a source for this data pattern, their assessment was validated by means of a phonetic analysis. Also, since listeners may perceive prominence patterns on syllable sequences in context even in the absence of definite acoustic cues for such a pattern (Dilley and McAuley, 2008), a validation of the raters' judgments seems appropriate. Hence, the syllable durations and pitch contours of sentences with perceived accented and unaccented *mehr* were compared.

**Figure 2:**
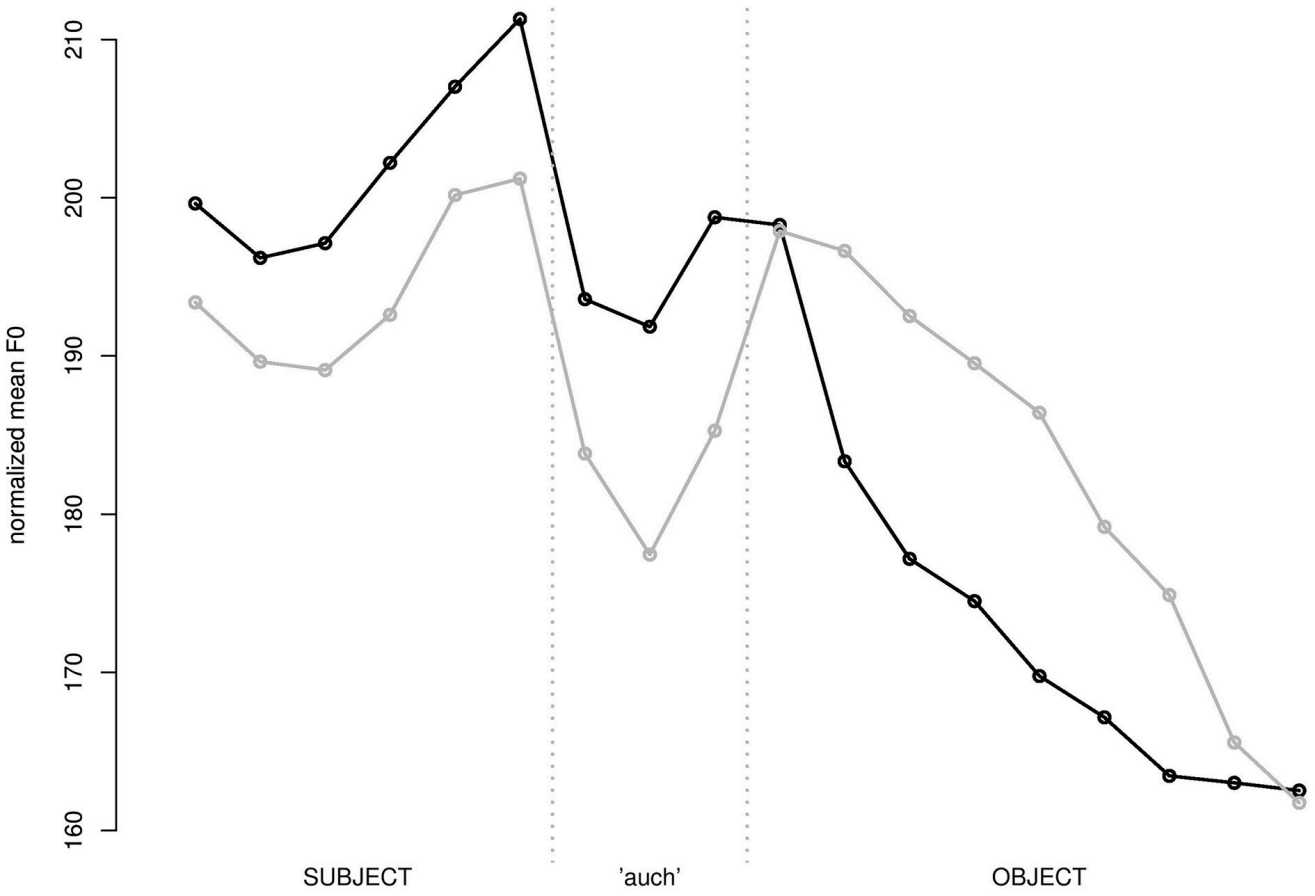
**Time normalized pitch contour (normalized mean pitch) for perceived accented (black) and unaccented (gray) versions of ***auch*****.

The 538 valid responses were annotated by a student assistant who was not informed about the purpose and the conditions of the experiment. Using the text-grid device in praat (Boersma and Weenink, 2010), the syllables in the critical region around *auch* were demarcated by hand, with two syllables preceding (corresponding to the subject of the embedded clause) and three syllables following *auch* (corresponding to the object). Each annotated syllable was split into three equal-sized intervals for which the mean F0 was recorded. The raw mean F0 values were normalized using the inverse of the utterance wide mean F0 multiplied by the global mean F0 (aggregated over speakers and items) as normalizing factor. The normalized values were interpolated to create an average time-normalized pitch contour. The plot in Figure 2 juxtaposes the time normalized contours for perceived accented (black) vs. perceived unaccented versions (gray) of *auch*. Apparently, accented *auch* (as revealed by a higher F0 on that word) coincides with a higher F0 rise on the preceding subject and deaccentuation of the object. These effects are perfectly in line with the expectations: *auch* is accented when it associates with subject focus, and focus may induce the higher prominence of the subject. In the same condition, the object is given, which may explain the deaccentuation on that constituent.

A linear mixed model evaluating the effect of accentuation on the (logarithmized) duration of *auch* confirms that it is significantly longer when it is perceived as accented (raw mean duration = 224 ms) than when it is not (raw mean duration = 197 ms) (Estimate: 0.126, Std.Err: 0.028, *t* = 4.47).

#### 2.2.4. Discussion

The oral reading experiment confirms that Context, preceding rhythm (RhythmLeft), and stress clash (RhythmRight) have (interactive) effects on the realization of accent on the ambiguous focus particle *auch*. The strong effect of Context on accentuation confirms that speakers do pay attention to the previous discourse when reading out the ambiguous target sentence. However, there is a high rate of context-target inconsistencies, especially for contexts inducing subject focus (only 40% of *auch* in subject focus conditions were perceived as accented). The high rate of inconsistencies shows that the task (unprepared oral reading) is appropriate to assess which reading is preferred in spontaneous reading without previous skimming. The clear preference for the object focus realization may be due to the fact that *auch* associating with subject focus may be expressed in a different way, i.e., with (unaccented) *auch* preceding the focused subject as in (6).

(6)        Carla glaubt, dass auch Herbert Lehrlinge überwacht.
               *Carla thinks that Herbert, too, supervises apprentices.*

*Auch* preceding the subject may in fact be a more natural expression of subject focus for three reasons: First, with this word order, there is no ambiguity as to the association of the focus operator—association of *auch* with the object is impossible / ungrammatical in (6). Secondly, in (6) but not in the subject focus versions of (5), the focus particle is left-adjacent to its scope domain. In this configuration, the focus particle acts as a herald for the focus domain—in contrast, postponed *auch* requires retrospective confirmation of the focus domain or, worse, reanalysis. The third reason is prosodic in nature: postponed *auch* associating with subject focus bears an accent (Féry, 2009); accent on function words, however, are highly marked^3^. Be this as it may, postponed *auch* is perfectly acceptable and grammatical in the subject focus contexts in (5).

The significant main effect of RhythmLeft confirms the hypothesis that the preceding trochaic beat—as established by the sequence of lexical prominences—leads to rhythmic expectations concerning upcoming material. As predicted, readers are less likely to accent *auch* when it falls onto an off-beat position or more likely to accent *auch* when it is in a strong position of the beat.

In contrast to our previous experiments (Kentner, 2012; McCurdy et al., 2013; Kentner, 2015), readers did not systematically avoid accentuation in the context of a potential stress clash to the right of *auch*—the effect of RhythmRight remains non-significant by itself. Rather, the significant three-way interaction shows that the effectiveness of the RhythmRight manipulation depends on the disposition of both RhythmLeft and Context. We will return to the lack of this effect in the General Discussion.

Under the assumption that the prosodic realization of *auch* reflects the readers' interpretation of the focus particle, we may submit that all three factors contribute to the way in which speakers interpret the target sentence.

However, experiment I does not allow firm conclusions to be drawn about the interplay of prosodic rhythm and contextual information in reading comprehension. So far, we have only evaluated data pertaining to speech production, which is known to lag behind interpretative processes in oral reading (Levin and Addis, 1979; Inhoff et al., 2011; Laubrock and Kliegl, 2015).

The eye-movement record provides data that is certainly more time-sensitive and thus more informative about the impact of implicit prosody and context in sentence comprehension (Clifton et al., 2007; Vasishth et al., 2013).

### 2.3. Experiment II: silent reading

Experiment 2 was an eyetracking version of Experiment 1.

#### 2.3.1. Methods

##### 2.3.1.1. Materials

The 24 item sets from Experiment 1 were again distributed over eight lists with items and conditions counterbalanced across the lists. Each list contained exactly one condition from each item set. In addition, 60 items from two unrelated experiments were interspersed as fillers. Each list was preceded by five practice items, yielding a total of 89 items per participant.

##### 2.3.1.2. Participants

Fifty-two native speakers of German from the Berlin area participated in the experiment for partial course credit or for payment. All participants reported normal or corrected-to-normal vision.

##### 2.3.1.3. Apparatus and procedure

Fixation time measures were gathered from the participants' right eye using an SMI (SensoMotoric Instruments) IView-X eye-tracker running at a sampling rate of 240 Hz (0.025 degree tracking resolution, and <0.5 degree gaze position accuracy). A chin rest was used to ensure stability. The chin rest was placed 55 cm from a 17 inch monitor (1024 × 768 pixel resolution). The angle per character was 0.3 degrees (3.8 characters per degree of visual angle). Stimulus presentation was controlled by Presentation software. Eye-gaze calibration was carried out at the beginning of the experiment, and calibration quality was monitored by the experimenter, with recalibration every ten trials, or more frequently if necessary.

Before each trial, the participant fixated upon a black dot in the center of the left side of the screen to ensure calibration quality. Successful fixation of the dot triggered the appearance of the context sentence, at which point the participant read it through and pressed a continuation button. The fixation point appeared once more at the same location, and after one second the point was replaced by the target sentence. Fixation data were gathered continuously throughout each trial. When the participant finished reading the sentence, either he or she was required to answer a yes/no comprehension question in the case of 36 out of the 60 filler items, or a fixation cross appeared on screen announcing the next trial. The two context sentences were broken into two lines, target sentences always appeared on one line. Participants took about 45 min to complete the experiment.

#### 2.3.2. Data analysis

The *em* package by Logačev and Vasishth (2006) was used to calculate the dependent measures from the raw output of the eye-tracking software. Inspection of the individual eye movement patterns revealed mis-calibration in three participants. Data from these participants were discarded, i.e., we considered data from 49 subjects.

##### 2.3.2.1. Regions of interest

We analyzed the eye movement data from four consecutive words, starting in the word preceding the focus particle *auch* up to the end of the sentence. The word preceding *auch* is the subject of the embedded clause and the locus of the RhythmLeft manipulation—if this word is disyllabic (trochaic), *auch* falls on a strong position of the the beat established by the preceding trochaic rhythm; conversely, *auch* is off beat relative to the established rhythm (i.e., on a weak position) in the case of a monosyllabic subject. At the same time, the subject is a potential bearer of the focus *auch* associates with. The second word of interest is the ambiguously attachable *auch*. The following object or, more precisely, the givenness or newness of the object, determines the interpretation of *auch*. When the object was already mentioned in the context, *auch* necessarily associates with subject focus and would bear nuclear accent in a spoken rendition of the sentence. In this case, the object would be de-accented. If the object was not previously mentioned, *auch* associates with object focus, with the object bearing the main sentence accent in a spoken rendition. The disambiguating object is also the locus of the RhythmRight manipulation. If starting in a stressed syllable, there is a potential stress clash if the critical *auch* would bear an (implicit) accent, which would be the case when *auch* is interpreted as associating with subject focus. The last word of the sentence is the verb of the subordinate clause. Irrespective of the experimental condition, this word is given in the discourse context.

##### 2.3.2.2. Reading measures

For the four regions of interest, we report three kinds of word reading times that were extracted from the eye-tracking data:

- First-pass reading time (FPRT, a.k.a. gaze duration): the summed duration of all fixations on a word before a fixation on any other word—given that no word to the right of the current word was fixated.
- Regression path duration (RPD) or Go-past time: summed duration of all fixations from the first fixation on the current word up to (but not including) the first fixation on a word further to the right. Note that this includes regressive fixations on words to the left of the current word.
- Total reading time (TFT): Summed duration of all fixations on a word

Fixations shorter than 50 ms were removed and treated as missing values. Fixation durations were log-transformed for inferential statistics.

#### 2.3.3. Results

Mean reading times and standard errors for the eight conditions are tabulated in Table 3. Linear mixed effects models (LMM, Bates et al., 2014) were employed to assess the influence of the fixed factors Context, RhythmLeft and RhythmRight, and their interactions on reading times. To ensure minimal correlations among the fixed effects, they were coded as orthogonal sum contrasts. Only the intercepts for participants and items were included as random effects. LMMs with a “maximal” random effect structure with varying intercepts and slopes, as advocated in Barr et al. (2013), did not converge or led to pathological estimates of the random effect correlations.

**Table 3:** **Raw reading times (FPRT, RPD, and TFT) with standard errors, broken down by condition, for the four critical regions (Subject of the embedded clause, focus particle ***auch***, Object, and sentence final Verb**.

**Context**	**RhythmL**	**RhythmR**	**FPRT (SE)**			
			**Subject**	***auch***	**Object**	**Verb**
Object foc	On beat	Unstressed	274 (19)	242 (16)	292 (21)	302 (26)
		Stressed	295 (22)	248 (16)	317 (26)	351 (29)
	Off beat	Unstressed	252 (15)	246 (16)	275 (19)	337 (31)
		Stressed	253 (15)	227 (15)	280 (23)	331 (28)
Subject foc	On beat	Unstressed	271 (18)	250 (16)	277 (20)	313 (24)
		Stressed	288 (24)	241 (15)	270 (19)	316 (33)
	Off beat	Unstressed	272 (20)	258 (15)	279 (19)	273 (22)
		Stressed	279 (19)	262 (16)	266 (21)	303 (25)
			**RPD (SE)**			
Object foc	On beat	Unstressed	317 (25)	279 (28)	419 (61)	692 (79)
		Stressed	333 (31)	313 (31)	430 (53)	654 (79)
	Off beat	Unstressed	302 (35)	268 (18)	365 (36)	672 (79)
		Stressed	310 (29)	266 (26)	408 (45)	649 (89)
Subject foc	On beat	Unstressed	339 (36)	265 (18)	387 (44)	587 (65)
		Stressed	354 (32)	283 (21)	367 (37)	623 (98)
	Off beat	Unstressed	323 (33)	282 (20)	389 (44)	644 (90)
		Stressed	318 (28)	322 (31)	388 (53)	659 (96)
			**TFT (SE)**			
Object foc	On beat	Unstressed	370 (29)	321 (33)	383 (34)	361 (28)
		Stressed	405 (40)	302 (22)	384 (36)	388 (35)
	Off beat	Unstressed	318 (29)	291 (21)	343 (29)	396 (35)
		Stressed	326 (24)	268 (21)	380 (37)	383 (29)
subject foc	On beat	Unstressed	365 (29)	285 (20)	338 (26)	344 (26)
		Stressed	378 (33)	282 (19)	336 (29)	353 (35)
	Off beat	Unstressed	330 (28)	314 (24)	353 (34)	355 (33)
		Stressed	325 (23)	313 (26)	324 (26)	391 (42)

In order to ensure that the results of the models with parsimonious random effect structure hold, we also fit Bayesian LMMs with the maximal random effect structure justified by the design of the experiment. In contrast to conventional LMMs, Bayesian LMMs always allow for complex random effect structures because the weakly informative priors used in the modeling will ensure that the posterior distribution of a parameter will be centered around 0 if there is not enough data to estimate the true value of the parameter. Since the results of the Bayesian analysis largely conform to the outcome of the conventional analysis, we report only the latter. The details of the Bayesian LMM are given in the Appendix.

**Figure 3:**
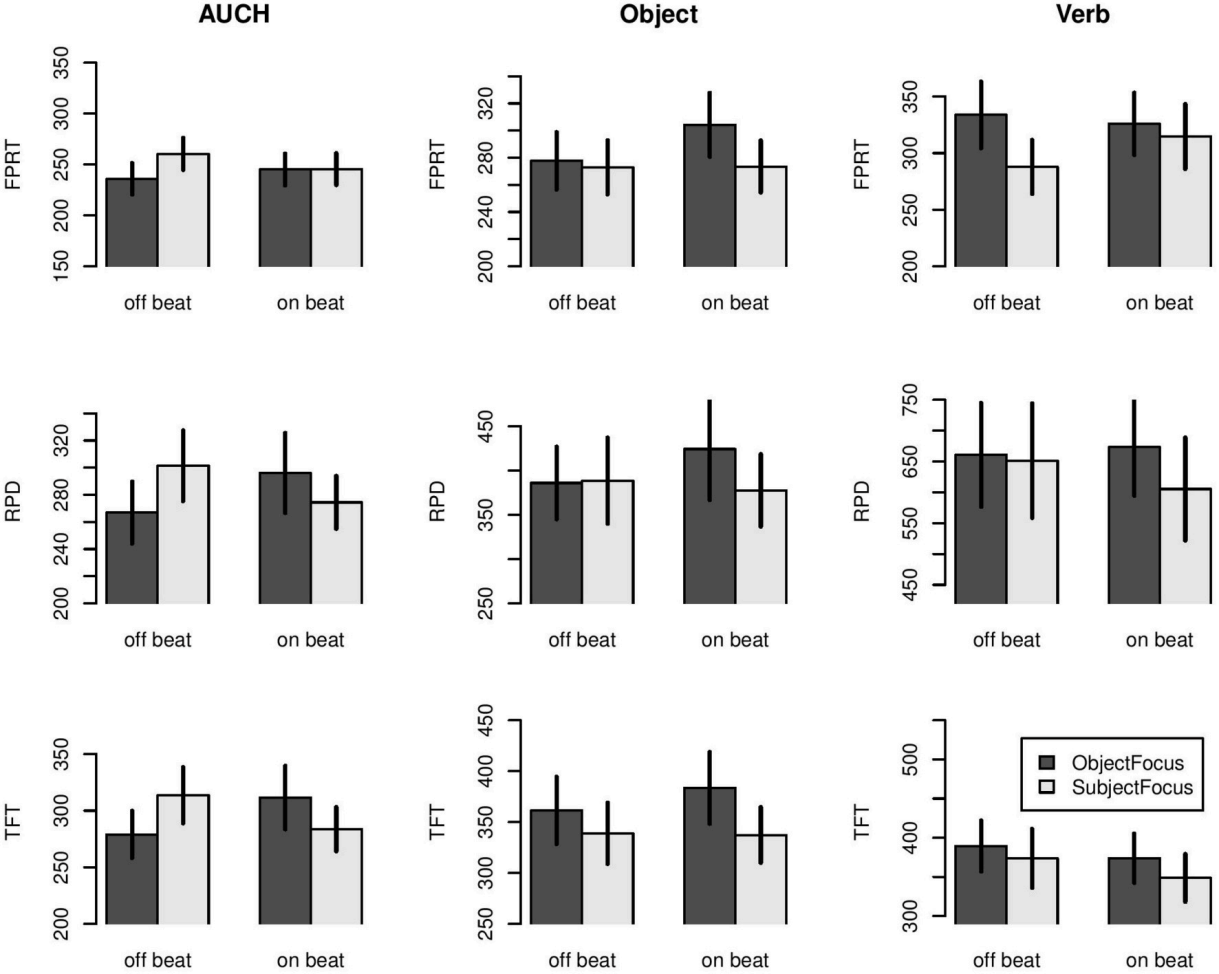
**First pass reading times (upper row), regression path durations (middle row) and total fixation times (lower row) for three consecutive words starting in the ambiguously attachable ***auch*** through to the sentence final verb**. Reading times are broken down by the factors Context (dark bars = Object focus, light bars = Subject focus) and RhythmLeft (*auch* off beat vs on beat). The factor RhythmRight is disregarded here. Error bars correspond to one standard error. Note that the contextual givenness of the Object determines the association of *auch*.

Figure 3 shows raw first pass reading times (upper row), regression path durations (middle row) and total fixation times (lower row) in milliseconds for three consecutive words starting in the ambiguously attachable *auch* through to the sentence final verb. The reading times are broken down by the factors Context (dark bars = Object focus, light bars = Subject focus) and RhythmLeft (*auch* off beat vs on beat). The factor RhythmRight is disregarded for reasons of clarity. The disambiguating object (middle column) determines the attachment site of *auch* and hence disambiguates the sentence.

We report inferential statistics on the reading data for the subject preceding *auch*, i.e., the locus of RhythmLeft manipulation, and the three following words.

##### 2.3.3.1. Subject preceding *auch*

All three reading measures reveal significant main effects of the RhythmLeft manipulation on the subject preceding *auch* (*t*-values≥|2|) with longer reading times for disyllabic (trochaic) subjects compared to monosyllabic ones. All other main effects and interactions are non-significant with *t*-values ≤ |1.2|, cf. Table 4.

**Table 4:** **Results of LMM for reading times on the Subject**.

**Subject**	**FPRT**		**RPD**		**TFT**	
	**Est.(SE)**	***t***	**Est.(SE)**	***t***	**Est.(SE)**	***t***
Context	0.007(0.01)	0.55	0.017(0.02)	1.06	−0.000(0.02)	−0.02
RhythmL	−0.026(0.01)	−2.00^+^	−0.034(0.02)	−2.2^*^	−0.071(0.02)	−4.46^*^
RhythmR	0.015(0.01)	1.13	0.016(0.02)	1.03	0.013(0.02)	0.85
Con×RhL	0.015(0.01)	1.20	0.002(0.02)	0.15	0.005(0.02)	0.35
Con×RhR	0.001(0.01)	0.04	0.002(0.02)	0.13	−0.007(0.02)	−0.46
RhL×RhR	−0.01(0.01)	−0.8	−0.006(0.02)	−0.38	0.001(0.02)	0.08
C×RL×RR	0.007(0.01)	0.55	−0.008(0.02)	−0.49	−0.003(0.02)	−0.2

*Statistics significant at the level of α = 0.05 (i.e., t-values≥|2|) are highlighted by an asterisk, or, if the 95% credible interval of the corresponding parameter in the Bayesian LMM includes 0, by a ^+^−sign*.

##### 2.3.3.2. Ambiguous *auch*

The eye-tracking data show reading times on *auch* to be affected by the factors Context and RhythmLeft (Table 5): FPRTs on *auch* are significantly longer when the Context manipulation requires subject focus.

**Table 5:** **Results of LMM for reading times on the focus particle ***auch*****.

***auch***	**FPRT**		**RPD**		**TFT**	
	**Est.(SE)**	***t***	**Est.(SE)**	***t***	**Est.(SE)**	***t***
Context	0.27(0.01)	2.14^+^	0.019(0.015)	1.27	0.012(0.02)	0.79
RhythmL	0.008(0.01)	0.67	−0.003(0.015)	−0.19	0.002(0.02)	0.19
RhythmR	−0.013(0.01)	−1.04	0.022(0.015)	1.48	−0.015(0.02)	−0.97
Con×RhL	0.025(0.01)	1.97	0.038(0.015)	2.53^*^	0.041(0.02)	2.66^*^
Con×RhR	0.006(0.01)	0.48	0.012(0.015)	0.79	0.011(0.02)	0.74
RhL×RhR	−0.008(0.01)	−0.62	−0.017(0.015)	−1.17	−0.014(0.02)	−0.9
C×RL×RR	0.016(0.01)	1.29	0.022(0.015)	1.48	0.004(0.02)	0.25

*Statistics significant at the level of α = 0.05 (i.e., t-values≥|2|) are highlighted by an asterisk, or, if the 95% credible interval of the corresponding parameter in the Bayesian LMM includes 0, by a ^+^-sign*.

In addition, RPDs and TFTs on *auch* reveal a significant interaction of Context and RhythmLeft to the effect that reading times on this word are longer when the position of the monosyllable *auch* with respect to the beat established by the preceding rhythmic context is in conflict with the contextually determined (implicit) accentuation or de-accentuation of *auch*. All other main effects and interactions on this word remain non-significant.

##### 2.3.3.3. Object following *auch* and sentence final verb

Reading times on the object (Table 6) reveal a main effect of context. FPRTs and TFTs are significantly longer in the object focus condition, i.e., for objects that were not mentioned in the previous context. Note that in the subject focus condition, participants already read the same object-verb sequence in the context. The same effects of context were found for FPRTs and TFTs on the sentence final verb (Table 7). In addition, there is a significant three-way interaction in FPRTs on the sentence-final verb, revealing a late influence of RhythmRight modulating effects of Context and RhythmLeft. RPDs on the object and the verb remain largely uninformative.

**Table 6:** **Results of LMM for reading times on the Object**.

**Object**	**FPRT**		**RPD**		**TFT**	
	**Est.(SE)**	***t***	**Est.(SE)**	***t***	**Est.(SE)**	***t***
Context	−0.397(0.19)	−2.08^+^	−0.026(0.02)	−1.43	−0.046(0.02)	−2.9^*^
RhythmL	−0.345(0.19)	−1.8	−0.02(0.02)	−1.09	−0.016(0.02)	−1.00
RhythmR	−0.054(0.19)	−0.01	0.002(0.02)	0.13	−0.000(0.02)	−0.02
Con×RhL	0.327(0.19)	1.71	0.016(.02)	0.91	0.016(0.02)	1.00
Con×RhR	−0.28(0.19)	−1.46	−0.028(0.02)	−1.56	−0.016(0.02)	−0.99
RhL×RhR	−0.165(0.19)	−0.86	0.003(0.02)	0.17	0.006(0.02)	0.34
C×RL×RR	0.024(0.19)	0.32	−0.006(0.02)	30.31	−0.011(0.02)	−0.7

*Statistics significant at the level of α = 0.05 (i.e., t-values≥|2|) are highlighted by an asterisk, or, if the 95% credible interval of the corresponding parameter in the Bayesian LMM includes 0, by a ^+^-sign*.

**Table 7:** **Results of LMM for reading times on the Verb**.

**Verb**	**FPRT**		**RPD**		**TFT**	
	**Est.(SE)**	***t***	**Est.(SE)**	***t***	**Est.(SE)**	***t***
Context	−0.041(0.02)	−2.38^+^	−0.045(0.02)	−1.87	−0.037(0.02)	−2.12^+^
RhythmL	−0.022(0.02)	−1.29	−0.001(0.02)	−0.05	0.01(0.02)	0.55
RhythmR	0.026(0.02)	1.51	−0.005(0.02)	−0.19	0.014(0.02)	0.77
Con×RhL	−0.014(0.02)	−0.79	0.017(0.02)	0.71	0.004(0.02)	0.24
Con×RhR	−0.011(0.02)	−0.63	0.018(0.02)	0.74	0.004(0.02)	0.22
RhL×RhR	−0.002(0.02)	−0.11	0.01(0.02)	0.39	0.001(0.02)	0.04
C×RL×RR	0.038(0.02)	2.2^*^	0.032(0.02)	1.33	0.022(0.02)	1.25

*Statistics significant at the level of α = 0.05 (i.e., t-values≥|2|) are highlighted by an asterisk, or, if the 95% credible interval of the corresponding parameter in the Bayesian LMM includes 0, by a ^+^-sign*.

### 2.4. Discussion

The data reveal an immediate if transient interaction of RhythmLeft and Context on *auch*, suggesting that readers consult and consider both sources of information simultaneously while forming an interpretation for the ambiguously attachable word. The eye tracking record attests enhanced reading effort if contextual and prosodic constraints on the interpretation of *auch* are in conflict. More concretely, reading times increase significantly when the monosyllable *auch* needs to be accented (subject focus) but falls onto a weak (off-beat), and hence less accentable, position with respect to the established rhythm.

In addition, the FPRTs show an early main effect of Context on *auch*. Note that both the main effect of Context and the interaction are in force before the disambiguating object has been fixated (as revealed by FPRT and RPD on this word). Since the contextual manipulation hinges on the givenness of the object directly following *auch*, the main effect in FPRT and the interactions of RhythmLeft and Context in FPRT and RPD suggest parafoveal preview of the object—given the shortness of *auch* this is a likely scenario.

Apart from the early Context×RhythmLeft interaction, the main effect of Context varies in polarity throughout the critical regions. On *auch*, the data suggest that readers experience more difficulty with subject focus readings. As noted above in Section 2.2.4, we assume that postponed *auch* in the subject focus reading is relatively marked and may therefore be the dispreferred reading. In contrast, on both the object and the verb, reading times are significantly shorter in the case of subject focus contexts. The reason for this disparity is likely due to the contextual givenness of the object in the subject focus conditions: in general, readers make shorter fixations on words that they have encountered shortly before. This familiarity advantage apparently overrides any effect stemming from the syntactic markedness of the subject focus condition.

The effect of RhythmRight is less systematic, and only becomes apparent in FPRTs on the sentence-final verb in the form of a three-way interaction. We discuss possible reasons for the weak influence of RhythmRight in the General Discussion.

## 3. General discussion

The two experiments reported here were designed to test the interaction of local phonological and more global, discourse-contextual information during the interpretation of structurally ambiguous sentences in oral and silent reading. In the first experiment (unprepared oral reading), we found a clear preference for the prosodic realization of the object focus reading with unaccented *auch*, a strong effect of Context (more accentuations of *auch* when the Context required the subject focus reading) and a weaker but systematic effect of RhythmLeft such that accentuation of *auch* was avoided in off-beat position. The effect of RhythmRight (avoidance of stress clash) turned out to be less systematic.

Similarly, the silent reading experiment yields an effect of Context, with reading times on the ambiguous word *auch* increased when the Context requires subject focus—we take this to confirm the general preference for the object focus reading that we found in the oral reading experiment. Moreover, a significant Context×RhythmLeft interaction on *auch* confirms that global discourse context and local prosodic rhythm conspire to condition the way the sentence is being interpreted. These effects were detected in so-called early reading time measures, which could suggest that they reflect early stages in the comprehension process (Clifton et al., 2007). Importantly, the Context×RhythmLeft interaction on *auch* emerges before readers fixated the disambiguating object. Therefore, the effect is unlikely to be driven by reanalysis processes; rather, it points to a guiding function of implicit rhythm in parsing, in line with findings by Breen and Clifton (2013) and Kentner (2012). Given the early influence of prosody on syntactic parsing, the present results are difficult to reconcile with accounts like the ones by Augurzky (2006), Kondo and Mazuka (1996), or Koriat et al. (2002), all of which consider syntactic structure building to be a prerequisite for the prosodic analysis in reading (see Kentner, unpublished, for a similar point).

What, then, is the nature of the early RhythmLeft×Context interaction affecting reading times on *auch*? Contextual givenness and low level linguistic rhythm are, at first sight, independent phenomena; an interaction may therefore seem surprising. While contextual givenness affects, even determines, the eventual association of the ambiguous focus particle *auch*, there is no obvious reason why the RhythmLeft manipulation—i.e., the variation of the syllabic structure of the subject preceding *auch* and, hence, the continuation of the established beat—should condition the interpretation of *auch*. However, the link becomes explicable when considering the prosodic consequences of the contextually determined interpretation of *auch*. Recall that a contextually given object induces *auch* to be associated with subject focus. In this case, *auch* bears the main sentence accent in a spoken rendition, and a corresponding implicit accent in silent reading. Conversely, if *auch* associates with object focus, it remains unaccented and the main accent is realized on the (newly introduced) object. The RhythmLeft manipulation engenders prosodic constellations that either facilitate or hinder accentuation of *auch*: If *auch* is “on beat” relative to the preceding trochaic rhythm (as established by the successive alternation of lexically stressed and unstressed syllables), accentuation is considered easy but it is considered hard when *auch* is “off beat.” The Context×RhythmLeft interaction reflect this: When, in order to comply with a contextual imperative, accentuation of *auch* is required but, at the same time, accentuation is hard on rhythmic grounds, readers tend to avoid accentuation in oral reading (Experiment I) or—in the case of silent reading (Experiment II)—the computation of the required structure is effortful and reading times increase. The results are therefore consistent with the early involvement of implicit prosodic rhythm and accentuation in written sentence comprehension. This interpretation is generally in line with our previous findings on the role of implicit prosody and rhythm in reading. However, there are notable differences concerning the details of the rhythmic and contextual effects, which we discuss below.

Kentner (2012) and McCurdy et al. (2013) explored the influence of linguistic rhythm on the interpretation of syntactically ambiguous structures like (7) in which the requirement for accentuation of the ambiguous word sequence *nicht mehr* depended upon its syntactic status as either a temporal adverb [(7-a), requiring unaccented *mehr*] or a negated comparative quantifier [(7-b), main phrase accent on *mehr*]. The rhythmic manipulation targeted the word following *nicht mehr*, featuring three-syllabic verbs with either initial or non-initial lexical stress.

(7)        Tim meint, dass man…
               Tim thinks that one…
              a.    nicht mehr {nachweisen / ermitteln) kann, wer der
                     not   more {determine / find out)     can,   who the
                     Täter  war.
                     culprit was.
              b.    nicht MEHR {nachweisen / ermitteln) kann, als
                     not   more     {determine / find out)    can,    than
                     die Zeit.
                     the time.

Both studies showed increased reading times for structures that engendered a stress clash (accented comparative *mehr* followed by a verb with initial stress) compared to non-clashing conditions. Kentner (2012) also reports an oral reading study in which readers avoid accentuation of the critical word when this leads to rhythmically infelicitous stress clash. While in Kentner's (2012) silent reading study, the effect of stress clash was detected at the disambiguating region following the verb, McCurdy et al. suggest that the rhythmic factor already affected the ambiguous word *mehr* itself. The rhythmic factor in those studies corresponds to the RhythmRight manipulation in the present experiments: i.e., the variation concerns the position of lexical stress on the word following a syntactically ambiguous, variably accentable word with the potential consequence of stress clash if the following word bears initial stress. However, comparing the results at hand with those by Kentner (2012) and McCurdy et al. (2013), it becomes clear that the present effect of RhythmRight on reading behavior deviates from the previous effects in that it is very limited and is detectable only relatively late.

A conceivable explanation for this disparity lies in the difference of the linguistic structures under study. As pointed out above, the structures of the present experiment (with *auch*) are superficially similar to the previously used items (with *nicht mehr*) in that the rhythm-syntax manipulation is brought about by a variably accentable, syntactically ambiguous word followed by a word featuring either initial or non-initial lexical stress. Despite this similarity, however, the syntactic relation of the ambiguous word with the following word differs between the experiments and experimental conditions: Consider first the case of the accented comparative quantifier *mehr* in (8-a): This word fills the object position of the following verb, and is thus part of the verb phrase, which, under standard assumptions, is mapped onto a prosodic phrase (Truckenbrodt, 2006). In contrast, accented *auch* in (8-b) is associated with the preceding subject and thus syntactically disjoint from the following object. A phrase boundary separating the two prominent syllables is a likely reason for the relatively limited effect of RhythmRight in this experiment—the boundary serves as a cesura that makes any effect of stress clash disappear (cf. Hayes, 1989; Sandalo and Truckenbrodt, 2003).

(8)        a.    …[ nicht mehr nachweisen kann]_*vp*_ als   …
                     …[ not    more   determine    can  ]_*vp*_ than…
               b.   … [ Hans  auch ]_*np*_ [ Lehrlinge    überwacht ]_*vp*_
                     … [ Hans, too,      ]_*np*_ [ apprentices supervises ]_*vp*_

Another difference between the present study and the experiment by McCurdy et al. (2013) concerns the effect of context and its interaction with the rhythmic manipulation. McCurdy et al. (2013) found only late effects of context and little interaction of context with prosodic rhythm. McCurdy et al. (2013) used a contextual priming strategy to bias the reader toward either the comparative or the temporal reading of ambiguous *nicht mehr*. There was, however, no compelling relation between the contextual bias and the resolution of the ambiguity in the target sentence. As opposed to such a loose relation between the context and the target ambiguity, the contextual manipulation of the present experiment is decisive for the correct interpretation of the ambiguous word—it hinges on the contextual givenness of the object. It may be the more compelling nature of the context sentence that led readers to take more careful note of its information when parsing the target sentence, resulting in earlier and stronger effects of context. A recent study by Logačev and Vasishth (2015) supports this view: building on work by Swets et al. (2008), Logačev and Vasishth (2015) show that contexts that are especially relevant for the interpretation of the target ambiguity may have important consequences for comprehension strategies as regards the target sentence. Specifically, they explored the nature of the ambiguity advantage that had been reported for globally ambiguous sentences (van Gompel et al., 2001, 2005), i.e., the fact that globally ambiguous sentences are read faster than non-ambiguous analogues. Logačev and Vasishth (2015) found that the presence of the ambiguity advantage depends on the nature of the comprehension questions readers were required to answer. While readers who had to answer superficial comprehension questions did show the ambiguity advantage, participants who had to respond to comprehension questions which required deeper text comprehension showed an ambiguity disadvantage, i.e., they read ambiguous sentences slower than the non-ambiguous counterparts. In the present eyetracking experiment, since readers were confronted with context sentences that are crucial for the appropriate interpretation of the target sentence, the context effect may be stronger and may have shown up early enough to directly interact with local prosodic information.

## 4. Conclusion

The two experiments presented in this study provide evidence suggesting that, during oral and silent reading, readers deploy both higher-level context and the rhythmic structure of German to disambiguate the attachment of the focus particle *auch*. A conflict between the disambiguation provided by context vs. rhythmic structure leads to a greater reading difficulty. We argue that such a conflict arises because both the contextual information, which co-determines the focus structure, as well as the prosodic rhythm, which establishes a prosodic prominence profile, affect the (implicit) accentuation of the text. This work therefore provides independent support for the claim that silent prosody plays an important role in parsing decisions, and that multiple sources of information are simultaneously deployed in resolving ambiguities.

## Author contributions

GK conceived Experiments 1 and 2, conducted Experiment 1, analyzed data of Experiments 1 and 2, wrote the manuscript. SV provided the eye-tracking infrastructure, analyzed the data of Experiments 1 and 2, wrote the manuscript.

### Conflict of interest statement

The authors declare that the research was conducted in the absence of any commercial or financial relationships that could be construed as a potential conflict of interest.

## Footnotes

^1^A fourth reading is available in which *auch* associates with verb focus, skipping a (given) object. The corresponding interpretation presupposes that H. is doing something to cellars in addition to rummaging through them. This reading is highly marked in that *auch* is not adjacent to the focus it associates with. Furthermore, it requires a prosodic rendition that deviates from normal phrasal stress in transitive VPs, i.e., with an unaccented object and nuclear accent on the verb. This reading is not viable in either of the contextual manipulations we devise in the experiment below and it will therefore be disregarded.

^2^Note that we cannot know what stage of the comprehension process exactly is reflected in the prosodic form of a read utterance because the articulation necessarily follows several, but presumably not all, interpretative processes in oral reading. It is very well possible that *auch* may be realized with accent although it was initially interpreted as associating with subject focus and vice versa. We assume here that accent on *auch* implies a preponderance of subject focus interpretation during processing and, conversely, unaccented *auch* implies a predominant object focus interpretation.

^3^In this context, it is interesting to note that the overall percentage of accents realized on *auch* in this experiment (25%) exactly corresponds with the percentage of perceived accented versions of the critical word *mehr* in the unprepared oral reading experiment in Kentner (2012), cf. example in (7).

## Appendix

In the analyses reported in Section 2.3.3, we used the lme4 package (Bates et al., 2014) and only fit varying intercepts models. This was because we could not fit maximal models due to convergence errors or boundary estimates of the correlation coefficients. In order to check that the effects hold up with a “maximal” model (Barr et al., 2013), we also fit Bayesian linear mixed models using the stan_lmer function in the rstanarm package (Gabry and Goodrich, 2016). It is almost always possible to fit a maximal model in the Bayesian setting because the weakly informative priors used in the modeling will ensure that the posterior distribution of a parameter will be centered around 0 if there is not enough data to estimate the true value of the parameter.

For the intercept, we used the prior *Normal*(0, 6^2^), and for the different comparisons, *Normal*(0, 1). For the full variance-covariance matrices (subjects as well as items) we defined an LKJ prior (Stan Development Team, 2014) on the correlation matrix; see Sorensen et al. (2015) for more details. One way to interpret the Bayesian LMM is to examine the 95% uncertainty intervals and, in addition, to calculate the probability from the posterior distribution that the parameter is less than 0 (*P*(*b* < 0). We will consider an effect to be present if the uncertainty interval doesn't contain 0. These effects are marked in bold in the tables below. The tables present modeling results for the three dependent variables (FPRT, RPD, TFT) in the four regions of interest (Subject, *auch*, Object, Verb), analogous to the conventional LMMs in Section 2.3.3.

**Table:**

**log FPRT Subject**					
	**Comparison**	**Mean**	**Lower**	**Upper**	***P*****(*****b*** < **0)**
1	Intercept	5.5048	5.4543	5.554	0
2	Context	0.0073	−0.0191	0.0336	0.2968
3	RhythmL	−0.0261	−0.0572	0.0062	0.948
4	RhythmR	0.0154	−0.0127	0.043	0.1403
5	Context:RhythmL	0.0154	−0.0118	0.0432	0.132
6	Context:RhythmR	7e-04	−0.0265	0.0274	0.4902
7	RhythmL:RhythmR	−0.0105	−0.0374	0.0163	0.768
8	Context:RhythmL:RhythmR	0.008	−0.0212	0.0383	0.2938
**log RPD Subject**					
	**Comparison**	**Mean**	**Lower**	**Upper**	***P*(*b* < 0)**
1	Intercept	5.63	5.5783	5.6853	0
2	Context	0.0163	−0.0159	0.0486	0.1698
3	RhythmL	**−0.0345**	**−0.0687**	**−5e-04**	**0.9778**
4	RhythmR	0.016	−0.0206	0.0531	0.1948
5	Context:RhythmL	0.0013	−0.034	0.0373	0.4702
6	Context:RhythmR	5e-04	−0.0329	0.0339	0.484
7	RhythmL:RhythmR	−0.0051	−0.0389	0.0293	0.6182
8	Context:RhythmL:RhythmR	−0.0081	−0.0426	0.0266	0.6742
**log TFT Subject**					
	**Comparison**	**Mean**	**Lower**	**Upper**	***P*(*b* < 0)**
1	Intercept	5.7148	5.6607	5.7683	0
2	Context	2e-04	−0.0356	0.033	0.4985
3	RhythmL	**−0.0701**	**−0.1063**	**−0.0336**	**1**
4	RhythmR	0.013	−0.0225	0.0486	0.2372
5	Context:RhythmL	0.0059	−0.0297	0.0408	0.3582
6	Context:RhythmR	−0.0081	−0.0434	0.0277	0.6768
7	RhythmL:RhythmR	9e-04	−0.0348	0.0349	0.4768
8	Context:RhythmL:RhythmR	−0.0025	−0.0376	0.0326	0.5645
**log FPRT** ***auch***					
	**Comparison**	**Mean**	**Lower**	**Upper**	***P*(*b* < 0)**
1	Intercept	5.4225	5.3736	5.4707	0
2	Context	0.026	−0.0056	0.0577	0.0552
3	RhythmL	0.0085	−0.0179	0.0354	0.2668
4	RhythmR	−0.0126	−0.0398	0.015	0.823
5	Context:RhythmL	0.0251	−0.002	0.0516	0.035
6	Context:RhythmR	0.0037	−0.0224	0.0301	0.3988
7	RhythmL:RhythmR	−0.0059	−0.0343	0.0214	0.6585
8	Context:RhythmL:RhythmR	0.0158	−0.0105	0.0426	0.119
**log RPD** ***auch***					
	**Comparison**	**Mean**	**Lower**	**Upper**	***P*****(*****b***<**0)**
1	Intercept	5.5262	5.4727	5.5812	0
2	Context	0.0177	−0.0171	0.0535	0.1595
3	RhythmL	−0.003	−0.0358	0.0301	0.5672
4	RhythmR	0.0237	−0.0081	0.0566	0.072
5	Context:RhythmL	**0.0387**	**0.0074**	**0.0705**	**0.0082**
6	Context:RhythmR	0.0106	−0.0216	0.0447	0.2648
7	RhythmL:RhythmR	−0.017	−0.0505	0.0164	0.8445
8	Context:RhythmL:RhythmR	0.0217	−0.01	0.0532	0.088
**log TFT** ***auch***					
	**Comparison**	**Mean**	**Lower**	**Upper**	***P*****(*****b***<**0)**
1	Intercept	5.5691	5.5142	5.624	0
2	Context	0.0123	−0.0216	0.0464	0.2238
3	RhythmL	0.0031	−0.0284	0.0348	0.4278
4	RhythmR	−0.0146	−0.0494	0.0207	0.7925
5	Context:RhythmL	**0.0412**	**0.0073**	**0.0744**	**0.0088**
6	Context:RhythmR	0.011	−0.0214	0.0428	0.241
7	RhythmL:RhythmR	−0.0133	−0.0473	0.0202	0.7852
8	Context:RhythmL:RhythmR	0.0044	−0.028	0.0365	0.3888
**log FPRT Object**					
	**Comparison**	**Mean**	**Lower**	**Upper**	***P*****(*****b***<**0)**
1	Intercept	5.519	5.463	5.5736	0
2	Context	−0.03	−0.0664	0.0081	0.9388
3	RhythmL	−0.0261	−0.0574	0.006	0.944
4	RhythmR	2e-04	−0.0352	0.035	0.489
5	Context:RhythmL	0.0259	−0.0056	0.0582	0.0548
6	Context:RhythmR	−0.0202	−0.0575	0.0168	0.8592
7	RhythmL:RhythmR	−0.0122	−0.0451	0.0207	0.7748
8	Context:RhythmL:RhythmR	0.0046	−0.027	0.0366	0.39
**log RPD Object**					
	**Comparison**	**Mean**	**Lower**	**Upper**	***P*(*b* < 0))**
1	Intercept	5.7581	5.6855	5.8276	0
2	Context	−0.0255	−0.07	0.0205	0.8742
3	RhythmL	−0.0197	−0.0607	0.0228	0.8258
4	RhythmR	0.0026	−0.0385	0.0451	0.4522
5	Context:RhythmL	0.0173	−0.0265	0.0632	0.2195
6	Context:RhythmR	−0.0291	−0.0693	0.0121	0.9192
7	RhythmL:RhythmR	0.0025	−0.0411	0.0461	0.4552
8	Context:RhythmL:RhythmR	−0.0052	−0.0453	0.0344	0.6022
**log TFT Object**					
	**Comparison**	**Mean**	**Lower**	**Upper**	***P*****(*****b***<**0)**
1	Intercept	5.7049	5.6316	5.7737	0
2	Context	**−0.0475**	**−0.0907**	**−0.0038**	**0.9802**
3	RhythmL	−0.0172	−0.05	0.0153	0.8408
4	RhythmR	−2e-04	−0.0396	0.0387	0.5062
5	Context:RhythmL	0.018	−0.0174	0.0538	0.1538
6	Context:RhythmR	−0.0151	−0.0556	0.0265	0.767
7	RhythmL:RhythmR	0.0042	−0.0367	0.0441	0.4232
8	Context:RhythmL:RhythmR	−0.0111	−0.048	0.0268	0.7185
**log FPRT Verb**					
	**Comparison**	**Mean**	**Lower**	**Upper**	***P*****(*****b***<**0)**
1	Intercept	5.5593	5.4736	5.6382	0
2	Context	−0.0384	−0.0797	0.0047	0.9618
3	RhythmL	−0.0161	−0.0549	0.0236	0.7892
4	RhythmR	0.0292	−0.0184	0.0782	0.113
5	Context:RhythmL	−0.0095	−0.0477	0.0269	0.6828
6	Context:RhythmR	−0.0092	−0.0516	0.0325	0.6548
7	RhythmL:RhythmR	0.0039	−0.0447	0.053	0.436
8	Context:RhythmL:RhythmR	**0.0416**	**0.001**	**0.0814**	**0.0225**
**log RPD Verb**					
	**Comparison**	**Mean**	**Lower**	**Upper**	***P*****(*****b***<**0)**
1	Intercept	6.1283	6.021	6.2321	0
2	Context	−0.0443	−0.0997	0.0121	0.94
3	RhythmL	0.0025	−0.0562	0.0632	0.4752
4	RhythmR	−0.0039	−0.06	0.0533	0.5495
5	Context:RhythmL	0.0181	−0.0345	0.0695	0.245
6	Context:RhythmR	0.0204	−0.0366	0.0766	0.2348
7	RhythmL:RhythmR	0.0107	−0.0448	0.0694	0.3618
8	Context:RhythmL:RhythmR	0.034	−0.0249	0.092	0.12
**log TFT Verb**					
	**Comparison**	**Mean**	**Lower**	**Upper**	***P*(*b* < 0)**
1	Intercept	5.7045	5.612	5.8004	0
2	Context	−0.035	−0.0758	0.0065	0.9535
3	RhythmL	0.0134	−0.034	0.0588	0.281
4	RhythmR	0.0145	−0.0293	0.0577	0.2455
5	Context:RhythmL	0.007	−0.0308	0.0478	0.3675
6	Context:RhythmR	0.0061	−0.0357	0.0495	0.4012
7	RhythmL:RhythmR	0.0049	−0.0431	0.0544	0.4145
8	Context:RhythmL:RhythmR	0.0233	−0.0181	0.0659	0.1365
